# Parents’, Teachers’, and Sledders’ Acceptability of a Virtual Reality Game for Sledding Safety Education: Cross-Sectional Study

**DOI:** 10.2196/63813

**Published:** 2025-05-21

**Authors:** Meggy Hayotte, Jonas Kreiner, Bernhard Hollaus

**Affiliations:** 1 Université Côte d'Azur, LAMHESS, France, Campus STAPS, 261 boulevard du Mercantour, Nice, 06200, France, 33 489153936; 2 Management Center Innsbruck (MCI), Department of Medical, Health & Sports Engineering, Innsbruck, Austria

**Keywords:** virtual reality, VR, sledding, safety education, Unified Theory of Acceptance and Use of Technology 2, UTAUT2, technology acceptability, cross sectional study

## Abstract

**Background:**

Sledding is a common recreational activity in the Alpine region, practiced by families, friends, and at school, but it is rarely considered to cause serious injuries. Current actions to promote safe sledding are limited to announcements at the start of dedicated tracks or to didactic sheets presented by teachers in schools. However, these actions are currently limited and do not allow the development of piloting technique skills. Virtual reality has the potential to develop piloting skills, although the development of a virtual reality for sledding safety education needs to be guided by its acceptability.

**Objective:**

The purpose of this study was to (1) examine the acceptability of the virtual reality game for sledding safety education from the perspective of different potential user profiles (ie, parents, teachers, and sledders) based on the Unified Theory of Acceptance and Use of Technology 2 enriched by the health locus of control (HLC), and (2) understand participants’ preferences and needs in terms of features to be integrated to the technology.

**Methods:**

Three profiles of participants (ie, parents, teachers, or sledders) were recruited through email diffusion from elementary schools, university, and ski resort announcements (Tyrol, Austria). They completed a series of questionnaires measuring: (1) demographic and general information, (2) acceptability of virtual reality sledding game for safety education (named VRodel), (3) perception of HLC, and (4) preferences and needs for VRodel.

**Results:**

A total of 122 participants (15 teachers, 43 parents or legal guardians, and 64 sledders) completed the survey. Findings reveal that the constructs of hedonic motivation (β=.43, *P*<.001), price sensitivity (β=.28, *P*<.001), and habit (β=.36, *P*<.001) explained 65% of the variance in behavioral intention to use VRodel. Two acceptability clusters were identified (low and high), but no differences between the acceptability clusters emerged based on age, status, gender, or previous virtual reality use. Internal HLC was positively correlated with all dimensions of acceptability except social influence and facilitating conditions. Some correlations between acceptability constructs were also shown with powerful others’ HLC. Participants highlighted the need to include realistic visual details and realistic interactions in the virtual environment for development.

**Conclusions:**

The acceptability of a virtual reality game for sledding safety education was quite high, and relationships with HLC were shown in the expected directions. Based on participants’ preferences, developers are advised to promote immersion in the game.

## Introduction

### Background

Sledding or tobogganing is a common recreational activity that is relatively low-cost and accessible, particularly developed in the alpine space (eg, Austria, Germany, or Switzerland). We can count more than 80 toboggan runs in Tyrol, Austria [1]. This physical activity, which consists of descending a mountain on a dedicated track with the use of a sled directed by a weight shift or by applying pressure on the runners, is widely practiced at all ages by families, friends, and at school during “snow class.” However, sledding is rarely considered to cause serious injuries and occasional death [2]. Hospital-treated tobogganing injury cases recorded by the Victorian Injury Surveillance Unit between 2004 and 2006 involved all age groups, from 5‐9 to 65‐69, with peaks in ages 10‐14, 20‐24, and 40‐44 years [3]. All age groups can therefore be considered to be concerned for potential sledding injuries. The report by the Swiss Council for Accident Prevention BFU (Beratungsstelle für Unfallverhütung), also highlights that all age groups are concerned by injuries, although 60% of injuries involve children and youth [4]. It is often assumed that sledding injuries occur mainly in children, however an analysis conducted in Switzerland over 13 years demonstrated that 45% of injuries were reported in the ≤20 age group, 33% in the 20‐40 age group, and 22% in the over-40 age group [5]. Consequently, the sledding injury prevention needs to address all age groups, which led us to consider adults first, as they can take on different roles as sledders, or as parents or teachers. As with road safety on bicycles, adults have an important role to play as parents and teachers in promoting safe sledding for children. The risk factors for sledding injury are (1) lack of hazard awareness and self-regulation, (2) lack of knowledge, and (3) insufficient skill in piloting techniques [4]. Developing self-regulation, knowledge, and skill in piloting techniques would promote better control of the sled, which would be a protective factor in avoiding injury. The need to develop sledding skills has become more pressing as the popularity tends to grow and the number of accidents increases [6]. Current actions to promote safe sledding are limited to announcements at the beginning of dedicated tracks, or didactic sheets presented by teachers in schools (eg, reports from the Swiss Council for Accident Prevention BFU). However, these actions are currently limited and do not allow the development of piloting technique skills.

### Virtual Reality for Unintentional Injury Prevention

Unintentional injury prevention (like sledding injury prevention) could be improved by the use of technology-based interventions. Among the technology used for injury prevention, virtual reality programs are the most commonly used [7]. In this systematic review, interventions targeted various populations (eg, children, parents, or school staff). The results highlighted that virtual reality programs have improved behaviors, but showed little gains in knowledge. Another systematic review on virtual reality for unintentional injury prevention training with children showed no significant differences in knowledge gained between virtual reality groups and control groups [8]. Therefore, we might expect that a virtual reality sledding game for safety education (VRodel) would not support knowledge development, but would be an effective strategy for improving an individual’s sledding skills, thus reducing the rate of luge injuries.

Virtual reality has been used in unintentional injury prevention to teach cognitive and motor skills (ie, decision-making or problem-solving skills, perceptual motor or psychomotor skills, procedural skills, and spatial skills) [8]. However, the transferability of these cognitive and motor skills to real-world environments was still mentioned as a critical gap in the literature [8]. Few studies have examined this gap nowadays. It has notably been shown in 1 study that similar behaviors were observed in real and virtual environments, even if speed was slightly overestimated in virtual environments [9]. Although virtual reality–based interventions are effective in developing skills in safety education contexts [7-9], it is necessary to examine them in the specific context of sledding safety education. However, before pursuing the long and expensive process of developing a virtual reality game for sledding safety, it is necessary to understand its potential reach. Although the emphasis needs to be on children and youth, all ages are concerned by sledding safety. It is therefore advisable to develop a game suitable for all potential user profiles (ie, sledders of any age, parents, or teachers). In particular, the acceptability of this type of technology and potential related factors needs to be examined in the light of recognized theoretical models.

### Acceptability of Virtual Reality

Technology acceptability refers to the a priori more or less positive mental representation that a user has before using a tool, which reflects their explicit willingness to use it [10]. Several models have been developed among theories of technology acceptability [10]. A recent systematic literature review on the acceptability of augmented reality in the field of training and education [11] highlighted that the Technology Acceptance Model [12,13], the Unified Theory of Acceptance and Use of Technology [14], and their variants are the most commonly used models in this context. Another systematic review that examined the acceptability of immersive virtual technologies in the context of exercise promotion in older adults failed to highlight theoretical models of acceptability [15]. In fact, in this review, the acceptability was reduced to an indicator of satisfaction. Satisfaction is considered as a determinant of use, which refers to the enjoyment of use [10]. The concept of hedonic motivation (HM), which is defined as “the fun or pleasure derived from using a technology” [16], is therefore close to the perceived satisfaction with the tool. As the Unified Theory of Acceptance and Use of Technology 2 (UTAUT2) [12] is today considered to be the most integrative, parsimonious, and predictive model of technology acceptability [17,18] and for its potential and previous uses in the context of virtual reality [11,19,20], we choose to adopt this theoretical framework.

The UTAUT2 combined eight theoretical models into seven constructs: (1) performance expectancy (PE), defined as “the degree to which using a technology will provide benefits to consumers in performing certain activities”; (2) effort expectancy (EE), defined as “the degree of ease associated with consumers’ use of technology”; (3) social influence (SI), defined as “the extent to which consumers perceive that important others (eg, family and friends) believe they should use a particular technology”; (4) facilitating conditions (FCs), defined as “consumers’ perceptions of the resources and support available to perform a behavior”; (5) price value, defined as the “consumers’ cognitive tradeoff between the perceived benefits of the applications and the monetary cost of using them”; (6) HM; and (7) habit (HT), defined as “the extent to which an individual believes the behavior to be automatic” [16]. These 7 constructs directly, or indirectly predict the future use of technology through behavioral intention (BI) [16]. Sex, age, and experience moderate these relationships in the model. For a new technology, without information on the actual price or the price of a similar technology, the relevance of the “price value” variable of the UTAUT2 could be questioned. Faced with this problem, Kapser and Abdelrahman [21] replaced “price value” by “price sensitivity” (PS). The PS, in the context of the UTAUT2, refers to the consumers’ willingness to pay for a specific technology.

Previous studies have recently applied the UTAUT2 to virtual reality in various contexts. For example, Boel et al [19] showed that PE, SI, FCs, and HM are significant predictors of BI of teachers to use immersive virtual reality in education. Bower et al [20], who studied the preservice teachers’ intention to use immersive virtual reality in education, revealed that UTAUT2 provided a suitable model in this context. They showed a wide variety of ratings among the UTAUT2 dimensions, with HM receiving the highest scores and HT scoring the lowest. It has already been critically argued in the literature that all the UTAUT2 constructs did not consistently predict BI to use a technology [22,23]. Therefore, the application of the UTAUT2 in the specific context of virtual reality for sledding safety education needs to be examined.

### Extensions of the UTAUT2

Several extensions have been made to the UTAUT2 into four directions: (1) new endogenous mechanisms, (2) new exogenous mechanisms, (3) new moderation mechanisms, or (4) new outcome mechanisms [23]. The UTAUT2 is robust and could be considered as a baseline model [23,24], however, other mechanisms could be considered regarding the specificities of the technology studied.

In the specific context of education and health promotion, we can consider the locus of control (LOC) or the health locus of control (HLC) as a potential extension of the UTAUT2. The HLC, originally conceptualized as a unidimensional construct, refers to the people’s belief that their health (or other events for the LOC) is or is not determined by their behavior, with at one end “health-externals” who believe they have little control, and at the other end “health-internals” who believe that their health (or other events for the LOC) is the result of their behavior [25]. Later, the concept was refined into three differentiated dimensions: (1) the internal HLC, which refers to an active role in one’s own health and taking responsibility toward health; (2) the powerful others external HLC, which refers to perceptions that other individuals, such as their physicians, control their health; and (3) the chance external HLC, which refers to the fact that health outcome is determined by luck, fate, and chance [26].

LOC has been previously reported to have relationships with acceptability constructs [27-29]. Internal LOC has been shown to be positively related to perceived usefulness, perceived ease of use, and perceived behavioral control in the context of learning [28,29]. Other authors [27] have shown that internal HLC was positively related to willingness to use health apps and willingness to use online trackers, as well as powerful others’ HLC. Meanwhile, another study [30], showed no direct relationship between HLC and BI to use mobile health, but an indirect relationship through PE, EE, and SI. However, to the best of our knowledge, no study has been conducted in the specific context of safety education, which would be particularly appropriate given the positioning of safety education as a cross between education and health promotion. Therefore, the relationships between HLC and the UTAUT2 need to be further investigated, especially in this specific context.

### Study Objectives and Hypotheses

This study will examine the acceptability of teachers, parents, and sledders of a virtual reality game for sledding safety education to guide the future development of the game. This study aimed to (1) characterize the acceptability of the virtual reality game for sledding safety education by parents, teachers, and sledders from the perspective of the UTAUT2-16 enriched by the HLC [26], and (2) understand participants’ preferences and needs in terms of features to be integrated to the technology.

A first series of hypotheses directly relied on the UTAUT2. As it has been demonstrated in the original model [16], it can be hypothesized that PE, EE, SI, FCs, HM, and PS would be positively related to BI to use the VRodel.

A second series of hypotheses was concerned with the relationships between acceptability constructs and HLC. Based on previous studies from the literature demonstrating positive relationships between the acceptability constructs and internal HLC [27-30]. Positive correlations are therefore expected in our study. Concerning the relationships between powerful others’ HLC, to the best of our knowledge, only 1 study demonstrated a positive correlation with willingness to use health apps and willingness to use online trackers [27]. As technology acceptability reflects users’ willingness to use a technology [10], we expected positive correlations between the UTAUT2 constructs and powerful others’ HLC.

## Methods

### Ethical Considerations

This study obtained ethical approval from the MCI (Management Center Innsbruck) Ethics Committee (number: 20230301). The administration of the questionnaires met the criteria of free participation, anonymity, and confidentiality of the responses, and was carried out online using LimeSurvey software (version 5.6.14; LimeSurvey, CE) with no possibility of missing data. All participants who voluntarily agreed to participate gave their electronic consent by clicking on “I agree to participate” in the online survey after having read the participant information sheet. The information sheet states that this acceptability study was conducted before the game’s development, and that the results will guide future development. No compensation was offered for completing the survey. Participants were informed that they can interrupt this study at any time for any reason without enduring any disadvantage. All survey responses were collected anonymously, and the data were stored on a secure server (MCI servers).

### Recruitment

All volunteer adults were eligible to participate in this study. Three participant profiles were sought: parents of children attending elementary schools; elementary school teachers; and sledders (individuals who go sledding at least once a year). Elementary schools in Tyrol (Austria) have been contacted by phone by JK to participate in the project by diffusing the link to the web-based questionnaire to parents and teachers of their schools. Additionally, posters were also distributed to ski resorts (Tyrol, Austria) to encourage participation in this study by sledders over the age of 18 years to ensure their free consent, as the study was only conducted online. The link to the survey was also publicly posted on social media by the research team members and project partners.

### Measures

#### Demographic and General Information

Participants completed a questionnaire gathering demographic data including age, gender, education level, current working status, their frequency per year of sledding, and their use of virtual reality. They were also asked to choose the perspective from which they were responding to the survey: (1) teacher, (2) parents or legal guardians, or (3) sledder.

#### Acceptability of VRodel

Participants read an illustrated written description of a game based on virtual reality for sledding safety education, named VRodel (Multimedia Appendix 1). After, they were invited to complete the following subscales of the German version of the UTAUT-2 scale [31]: PE (4 items), EE (4 items), SI (3 items), FCs (4 items), HM (3 items), HT (4 items), and BI (3 items). Given the current development of the VRodel proof of concept, no price could be set in the description. Therefore, we replace the price value by the PS, as done by Kapser and Abdelrahman [21]. The PS was measured through 5 items [21,32]. All items were measured on a 7-point scale ranging from 1=strongly disagree to 7=strongly agree.

The tense of the items was changed from present to conditional, as VRodel was only a proof of concept, and to our knowledge, no similar technology was commercially available. Items were slightly adjusted to the specific context of sledding, and “Pokémon Go” was replaced by “VRodel.” Therefore, we pretested the clarity of the material. The clarity of the VRodel description was assessed with 2 items (ie, “The VRodel description is clear and understandable”; “The description allows me to project myself in the use of VRodel”), and 1 item to examine the need for illustration (ie, “I will need an illustration to better project myself in the use of VRodel”) on a 7-point Likert scale from 1=strongly disagree to 7=strongly agree. Participants rated the modified instrument by scoring the clarity (ie, “to what extent do you think the statement is clear and understandable?”) of each item on a 7-point Likert scale ranging from 1=strongly disagree to 7=strongly agree.

Participants were also asked to justify and propose a rewording of low-rated items through comments. A first clarity survey was conducted with a convenience sample of 13 participants (4 females, 9 males; mean age (M_age_) 25.6, SD 3.8 y). Results showed acceptable clarity scores (mean 5.3/7, SD 0.7). However, 11 items were below 5/7 and were rephrased. Even the VRodel description was clear and allowed the projection into usage (mean 6/7, SD 1), based on participants’ comments, we have slightly rephrased the VRodel description and added a VRodel illustration (ie, requested at mean 6.4/7, SD 0.9). A second clarity survey was conducted with a sample of 11 participants from the target population (7 females, 4 males; 6 sledders, 3 teachers, and 2 parents; M_age_ 30.7, SD 9.0 y). The results showed acceptable clarity scores (mean 5.9/7, SD 0.5). All items were above 5/7 except 1 item (ie, HT3). This item was rephrased and examined in a third clarity survey with a sample of 11 participants from the target population (2 females, 9 males; 8 sledders, 2 teachers, and 1 parent; M_age_ 27.3, SD 6 y) and scored a mean of 6.2/7 (SD 0.8).

Because several adaptations were made, we examined the factor structure of the scale. The bifactorial confirmatory model with 27 items showed a good fit to the data: *χ*^2^_277_=425.23, *P*<.001, CFI=.92, TLI=.90, RMSEA=.067. Cronbach α ranged from 0.76 to 0.91 and were considered good [33], except for HT (α=0.45), which is lower and should be considered with caution.

#### Perception of Health Prevention

The German HLC questionnaire [34] was completed by participants. This scale contains three dimensions: (1) internal HLC (7 items), (2) powerful others’ HLC (7 items), and (3) chance HLC (7 items). All items were measured on a 7-point scale ranging from 1=strongly disagree to 7=strongly agree. Cronbach α ranged from 0.69 to 0.77 and were considered reasonable [33].

#### Preferences and Needs for the Development of VRodel

Some questions were designed to measure preferences and needs for VRodel to define future development directions. Participants were asked for different locations (eg, school, ski resorts, hotels or lodges, tourist information centers, or at home) and to what extent they think VRodel should be implemented, on a scale from 1=strongly disagree to 7=strongly agree. Needs in terms of virtual reality immersive experience in VRodel (eg, realism or gamification) were also asked of participants to define priority development directions. Twelve affirmations were designed by the team members involved in the VRodel project: (1) realistic visual details (trees, terrain, etc); (2) realistic sounds; (3) simulation of accidents; (4) increase of vibrations with increasing speed; (5) ideal driving line marked as arrows or lines; (6) game elements (rewards, badges, tokens on the track to collect, etc); (7) information about safe behavior on the toboggan run (how to deal with other people, proper sitting position while tobogganing, or how to get up to the toboggan run); (8) other sledders who are also on the same toboggan run; (9) multiplayer mode with interaction with other players; (10) race mode (high scores based on your own time); (11) bonus items (speed enhancers or traction enhancers); (12) changes in visual details (night mode, fog, or poor visibility). For each item, participants were asked to rate on a 7-point scale ranging from 1=strongly disagree to 7=strongly agree their level of agreement with the following statement: “I think VRodel should include…”

### Statistical Analysis

Analyses were performed using SPSS AMOS (version 23; IBM Corporation). The data was first checked to remove unusable surveys, and missing data was analyzed. The skewness ranged from −1.2 to 2, and the kurtosis ranged from −1.1 to 4, which can be considered a normal distribution [35].

Multiple regression analyses were used to examine the explained variance and the main contributors to BI to use VRodel. A cluster analysis, based on the K-means method, was then conducted to examine the acceptability groups. This approach has been used in several studies for similar purposes [36]. The optimal number of groups was determined based on the elbow method [37] and with a principle of parsimony. Following this method, 1 to 5 clusters were iterated, and the within-cluster sum of squares (ie, distance from the centroid of the cluster) was calculated for each k-cluster. Then, the graph representing the within-cluster sum of squares on the y-axis and the k values (ie, number of clusters) on the x-axis was plotted. The within-cluster sum of squares decreases by adding more clusters. The optimal number of clusters was determined by identifying the k-cluster, before adding a new cluster, which results in only a marginal decrease in the within-cluster sum of squares. We labeled the groups regarding their characteristics of acceptability of VRodel. ANOVA was performed to assess potential age differences between groups. Chi-squared tests were used to assess potential differences in status (ie, parents, teachers, or sledders), gender, and previous virtual reality use between groups.

Correlations between acceptability constructs and HLC dimensions were computed. Then, a multivariate ANOVA was conducted to examine whether HLC differed between acceptability groups.

Participants were also invited to express their preferences and needs for VRodel through specific questions. These questions were summarized using descriptive statistics and presented according to the acceptability of VRodel clusters, if appropriate.

As the main analyses were the examination of relationships, the rule of thumb recommended a minimum of 50 participants [38]. For multivariate analyses, larger sample sizes are recommended. Therefore, a minimum of 100 participants was expected.

## Results

### Participants’ Characteristics

Data was collected from March to May 2023. A total of 163 surveys were completed, of which 41 were excluded (ie, refusal of consent, n=3; incomplete survey without at least the items of acceptability of VRodel completed, n=26; abnormally short or long time to complete the survey, n=1; or individuals with the role of sledder with not at least 1 report of sledding per year, n=11).

The final sample of 122 participants (Table 1) was composed of 15 teachers (10 females, M_age_ 41.7, SD 11.2 y), 43 parents or legal guardians (23 females, M_age_ 46.6, SD 12.4 y), and 64 sledders (18 females, M_age_ 28.8, SD 16.6 y). Missing data (ie, HLC) are present for 12 individuals who stopped completing the survey before the end but they were considered in analyses.

**Table 1.** Characteristics of participants evaluating the acceptability of a virtual reality game for sledding safety education (N=122).

Variable and level		Value
Gender, n (%)		
	Male	71 (58.2)
	Female	51 (41.8)
Age (years), mean (SD)		36.8 (14.5)
Participant’s profile, n (%)		
	Parent or legal guardian	43 (35.2)
	Teacher	15 (12.3)
	Sledder	64 (52.5)
Employment status, n (%)		
	Full-time employed	55 (45.1)
	Part-time employed	19 (15.6)
	Retired	7 (5.7)
	Studying	41 (33.6)
Level of education (years), n (%)		
	<12	14 (11.5)
	12	49 (40.2)
	15	30 (24.6)
	≥15	29 (23.8)
Frequency of sledding, n (%)		
	≤1 time per year	36 (29.5)
	2 to 4 times per year	44 (36.1)
	5 to 9 times per year	17 (13.9)
	≥10 times per year	25 (20.5)
Frequency of virtual reality use, n (%)		
	Never	30 (24.6)
	Sometimes	70 (57.4)
	Often to very often	22 (18.0)

### Acceptability of VRodel

Acceptability constructs were rated as moderate to high (M_PE_ 4.32, SD_PE_ 1.29; M_EE_ 5.01, SD_EE_ 0.84; M_SI_ 3.77, SD_SI_ 1.40; M_FC_ 4.31, SD_FC_ 4.08; M_HM_ 5.22, SD_HM_ 1.24; M_BI_ 3.64, SD_BI_ 1.35), except for HT and PS which are perceived quite low (M_HT_ 2.40, SD_HT_ 1.24; M_PS_ 2.74, SD_PS_ 1.13). The acceptability constructs explained 65% of the variance in BI to use VRodel (*F*_7,114_=32.82, *P*<.001). HM (β=.43, *P*<.001), HT (β=.36, *P*<.001), and PS (β=.28, *P*<.001) are significant contributors to the BI to use VRodel. Other acceptability constructs (ie, PE β=−.05, *P*=.53; EE β=.06, *P*=.36; SI β=.10, *P*=.21; FCs β=−.03, *P*=.62) were not significant contributors to the BI to use VRodel.

**Figure 1.**
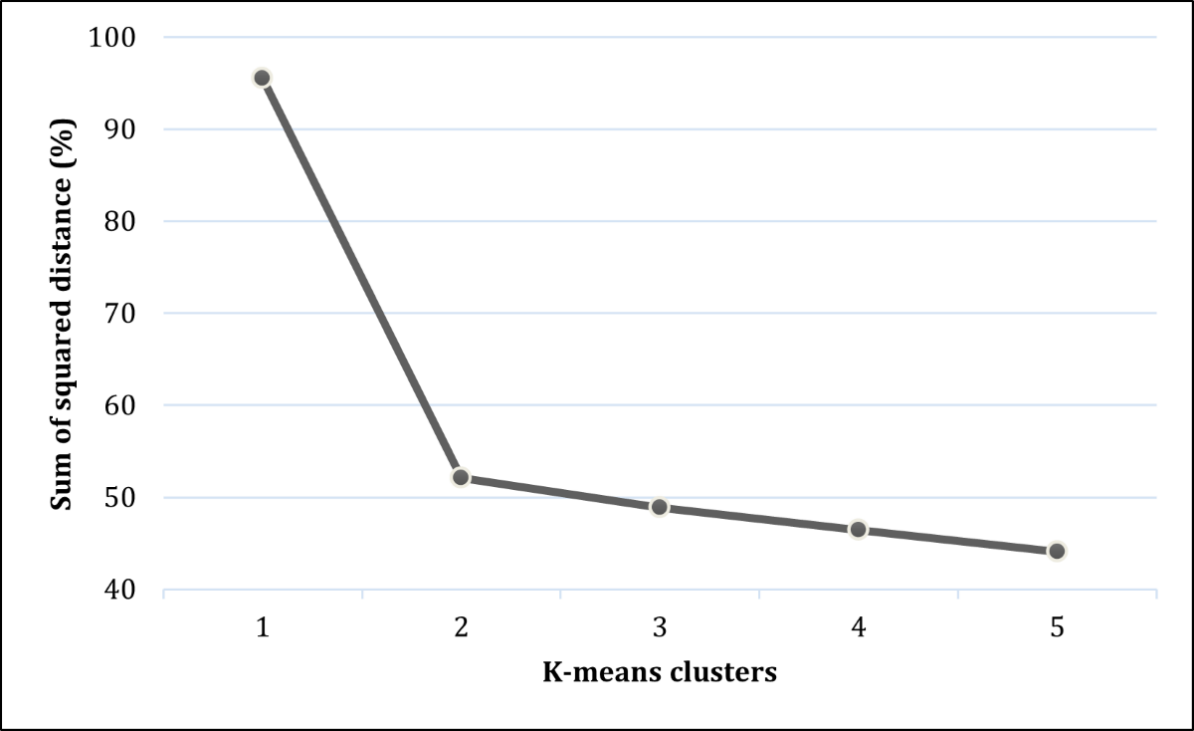
Within-cluster sum of squares reduction (percentage) for k-means clusters (ranging from 1 to 5) of acceptability of a virtual reality game for safety sledding education.

**Figure 2.**
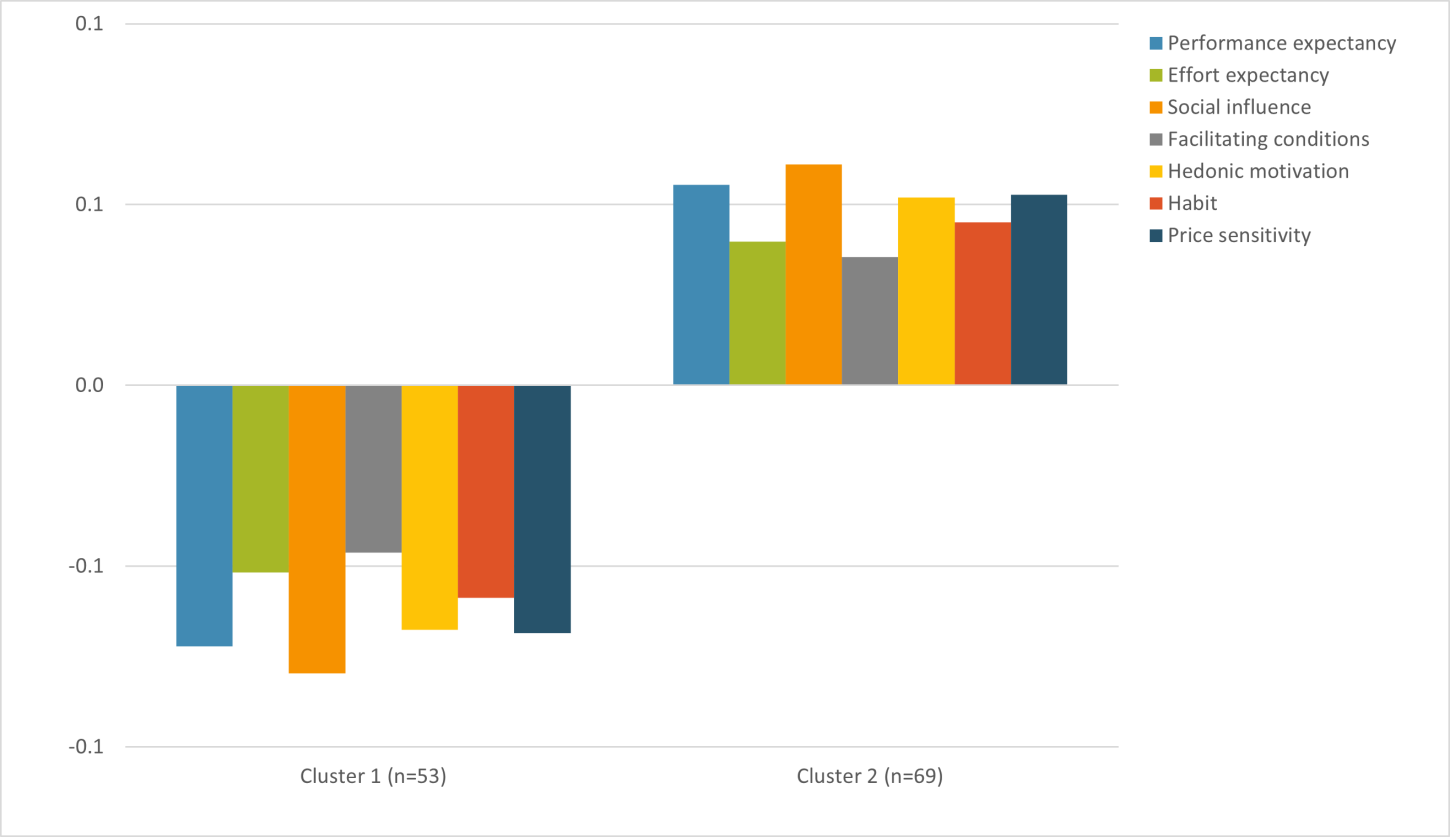
Z-scores of the acceptability of a virtual reality game for safety sledding education constructs in the low-level cluster (1; n=53) and the high-level cluster (2; n=69).

According to Figure 1, we can see an “elbow” [37] for the 2-cluster option. This solution was selected, and the clusters were examined for each acceptability dimension. The first cluster, composed of 53 participants, is characterized by low levels of all acceptability constructs, whereas the second cluster, composed of 69 participants, is characterized by high levels of all acceptability constructs (Figure 2). ANOVA showed no age differences between the acceptability clusters (*F*_1,120_=0.98, *P*=.32). Chi-squared tests showed no difference between clusters for status (*χ*²_2_=0.79, *P*=.67), gender (*χ*²_1_=1.37, *P*=.24), or previous virtual reality use (*χ*²_2_=0.60, *P*=.74).

### Relationships Between Acceptability of VRodel and HLC

Internal HLC was positively correlated to PE (*r*=0.29, *P*=.003), EE (*r*=0.30, *P*=.002), HM (*r*=0.31, *P*=.001), HT (*r*=0.25, *P*=.01), PS (*r*=0.20, *P*=.04), and BI to use VRodel (*r*=0.27, *P*=.004). Powerful others’ HLC was positively correlated to PE (*r*=0.30, *P*=.003), SI (*r*=0.31, *P*=.001), HT (*r*=0.41, *P*<.001), PS (*r*=0.38, *P*<.001), and BI to use VRodel (*r*=0.22, *P*=.02). Chance HLC was positively correlated to HT (*r*=0.22, *P*=.02).

A 1-way multivariate ANOVA revealed a main effect of cluster membership on HLC (Wilks Ʌ=.86, *F*_3,106_=5.96, *P*=.001, η_p_^2^=.14). Participants of the 2 acceptability of VRodel clusters exhibited differences in terms of internal HLC (*F*_1,108_=7.17, *P*=.009, η_p_^2^=.06) and powerful others’ HLC (*F*_1,108_=12.89, *P*<.001, η_p_^2^=.11). Participants in the low acceptability of VRodel cluster (n=47) tend have lower levels of internal HLC (M_Internal HLC_ 4.45, SD_Internal HLC_ 0.76) and powerful others’ HLC (M_Powerful others’ HLC_ 3.47, SD_Powerful others’ HLC_ 0.78) than participants in the high acceptability of VRodel cluster (n=63; M_Internal HLC_ 4.85, SD_Internal HLC_ 0 .79; M_Powerful others’ HLC_ 3.98, SD_Powerful others’ HLC_ 0.71). No differences were found between clusters for chance HLC.

### Preferences and Needs for the Development of VRodel

Regarding perceptions of where VRodel should be integrated, no differences emerged between the acceptability clusters, therefore, the results were presented for the overall sample of participants. VRodel implementation locations have been ranked in the following order: hotels or lodges (mean 5.64/7, SD 1.07), tourist information centers (mean 5.35/7, SD 1.42), ski resorts (mean 5.13/7, SD 1.64), at home (mean 4.76/7, SD 1.73), and at school (mean 4.28/7, SD 1.76).

Regarding the priority of features to be implemented in VRodel, some differences emerged between the clusters, therefore, the results were presented for each cluster (Table 2). All features presented to the participants scored higher than 4/7, except for the bonus items in the low acceptability of VRodel cluster, meaning that all features could be interesting to implement. However, realistic visual details, information about safe behaviors, and realistic interactions in the virtual environment through speed-dependent vibration were the top 3 features requested by all participants.

**Table 2.** Evaluation of the features to implement in VRodel from the high- and low-level clusters of acceptability of a virtual reality game for safety sledding education.

Features to implement in VRodel	Low-level cluster of acceptability of VRodel^a^ (n=53), mean (SD)	High-level cluster of acceptability of VRodel^a^ (n=69), mean (SD)	Priority
Realistic visual details	6.2 (1.2)	6.4 (1)	1
Information about safe behavior on the toboggan run	6.1 (1.2)	6.3 (1.1)	2
Increase in vibrations with increasing speed	5.8 (1.2)	6.3 (0.9)	3^b^
Changes in visual details (night mode, fog, or poor visibility)	5.7 (1.3)	6.1 (1.2)	4
Realistic sounds	5.4 (1.4)	6 (1.2)	5^b^
Other tobogganers who are also on the same toboggan run	5.7 (1.2)	5.8 (1.3)	6
Simulation of accidents	5.5 (1.5)	5.8 (1.3)	7
Ideal driving line marked as arrows or lines	5.4 (1.4)	5.7 (1.2)	8
Game elements (rewards, badges, tokens on the track to collect, etc)	4.9 (1.9)	5.6 (1.3)	9^b^
Multiplayer mode with interaction with other players	5 (1.7)	5.5 (1.4)	10
Race mode (high scores based on your own time)	5.2 (1.6)	5.3 (1.7)	11
Bonus items (speed enhancers and traction enhancers)	3.6 (1.8)	4.3 (1.8)	12^b^

a Scores are expressed on a 7-point scale ranging from 1=strongly disagree to 7=strongly agree and presented as the mean and SD for each cluster.

b Significant difference (*P*<.05) between the two clusters.

## Discussion

### Principal Results

This study carried out an important first step in the development process of a new tool, the evaluation of its acceptability. A strength of our study is that acceptability characterization was based on strong theoretical foundations with the perspective of the UTAUT2 [16] enriched by the HLC framework [26]. The results showed moderate to high scores of acceptability, except for HT and PS, which are perceived as quite low. HM was the construct of acceptability rated as the highest, whereas HT was the lowest. HM (β=.43, *P*<.001), HT (β=.36, *P*<.001), and PS (β=.28, *P*<.001) were significant predictors of BI to use VRodel (65% of explained variance). These results suggest that VRodel should be well accepted, as long as it will integrate gamified components, will not be too expensive, and will be based on the actual low virtual reality usage of future users. Two clusters emerged with low and high levels of acceptability, respectively. No differences were found between the characteristics of participants in the 2 clusters (ie, age, participant’s profile, gender, or previous use of virtual reality). Therefore, it seems that a common game could be first drafted for all users and then declined depending on its effectiveness, which would be examined in a future study.

Internal and powerful others’ HLC were higher in the high acceptability of the VRodel cluster than in the low acceptability of the VRodel cluster. The acceptability of a virtual reality game for sledding safety education constructs was correlated with internal and powerful others’ HLC. To the best of our knowledge, this is the first study to demonstrate relationships between HLC and UTAUT2 for virtual reality in an educational setting. It is therefore important to note that for virtual reality with a health educational purpose, the HLC is suitable to enrich the UTAUT2.

“Realistic visual details” was the most requested feature. This feature can be achieved through photogrammetry. The use of photogrammetry for the design of virtual environments is not novel [39], but it tends to become more widespread for sports purposes [40]. It is possible to create a virtual world that is perceived as real from the ground up, but it is necessary to record high-quality video with drones (eg, Phantom 4 or Mavic 3 from Da Juang Innovation [DJI] Enterprise). The position measurement of the drone could be improved by using a ground station. This technique could also be combined with the Unity Terrain Tools software. All features of VRodel have been well evaluated, so we can recommend the integration of all of them according to their priority level established in this study. Finally, the implementation of VRodel was not positioned primarily for diffusion and integration in schools, as team members had assumed. In fact, the school appeared to be the least appropriate place to implement this technology. It would thus be appropriate to work further with tourist sites (ie, hotels or lodges, tourist information centers, or ski resorts) to establish the technical specifications for the development of the VRodel game. The findings of this study have significant implications to help developers and engineers build an immersive virtual reality game for sledding safety education.

### Limitations

This study nevertheless has some limitations. First, participants only read an illustrated written description of a game based on virtual reality for sledding safety education. The description was short which could lead to unrealistic expectations for the game. Therefore, the acceptability scores cannot be generalized, and another study of acceptability with actual testing of the game would be necessary.

Another limitation is the cross-sectional design of this study, where all measures were collected at the same time. Methodological precautions were taken by using validated questionnaires in a specific order, and testing of their validity and reliability after small modifications. Participation in this study was voluntary with no incentive, and slow responses were excluded, so we could expect a sufficient level of motivation from participants to complete the online survey. However, a common method bias could still occur [41]. In future studies of the acceptability of VRodel with actual testing of the game, it would be interesting to obtain measures of the predictor and criterion variables from different sources and including a temporal separation between them [41].

Although no differences emerged between the different profiles of participants, we recognize that the number of teachers was small. Additional studies would be needed to better understand teachers’ perceptions if VRodel was implemented in school for teaching sledding safety (although this type of implementation was not recognized as a priority). The representativeness of the sample is therefore limited, and although the results have led to technical specifications for the development of a functional VRodel prototype, it would be necessary to continue involving future potential users with different profiles in the following stages of VRodel development.

### Comparison With Prior Work

Hypotheses that acceptability constructs would predict the BI to use VRodel, as per the original model [16], were partially validated. Our study demonstrated that only HM, HT, and PS were significant contributors to the BI to use VRodel. Therefore, in this specific context of virtual reality for safety sledding, not all acceptability constructs predict BI. This is in line with other studies demonstrating the differences regarding the specificities of the contexts and technologies considered [22,23]. Per Boel et al [19] and with Bower et al [20] in the context of immersive virtual reality in education, HM also predicts BI. PS, which is related to price value, was shown to be a predictor of BI, as in the study by Tseng et al [42]. Although HT was found to be a predictor of BI, no differences emerged in terms of previous experience with virtual reality between the acceptability of VRodel clusters. Surprisingly, PE was not a predictor of BI as in other similar studies [19,42]. A potential explanation could be the lack of knowledge about the effectiveness of virtual reality for safety education, resulting from the presentation of a proof of concept of the VRodel to be developed, rather than the real game with prior interaction with it. Few other studies have already shown no relationship between PE and BI in the education context [43]. It would be necessary to further investigate this relationship after usage of the game.

Internal and powerful others’ HLC were positively correlated to certain constructs of the UTAUT2, in line with our hypotheses. In line with Bennett et al [27], internal and powerful others’ HLC were positively correlated with BI to use VRodel. Our results were also partially in line with Ahadzadeh et al [30] and showed that PE, EE, and SI were related to HLC. Although no relationship was expected between chance HLC and UTAUT2 constructs, a small correlation was found between HT and chance HLC. This result should be interpreted with caution, given that the internal consistency of the HT subscale was rather low (ie, α=0.45). While the relationships between UTAUT2 and the 3 subscales of HLC have been poorly investigated in the literature, these findings are congruent with other similar studies [27-29].

### Conclusions

This study characterized the acceptability of the virtual reality game for sledding safety education by parents’, teachers’, and sledders’ perspectives by applying the UTAUT2 in conjunction with the HLC framework. It is therefore the first study to demonstrate that HLC is suitable for enriching the UTAUT2 in the context of virtual reality for unintentional injury prevention. Important directions for the effective development of a virtual reality game for sledding safety education have been highlighted and prioritized. Future studies would be needed to examine the effectiveness of such a technology and the transferability of knowledge and skills acquired in a virtual environment to the real-world environment of a sled track.

## Supplementary material

10.2196/63813 Multimedia Appendix 1 Original German version and translated English version description of the virtual reality sledding game for safety education.

10.2196/63813 Checklist 1 RATE-XR checklist. RATE-XR: Reporting for the Early-Phase Clinical Evaluation of Applications Using Extended Reality.

## Abbreviations

BFU: Beratungsstelle für Unfallverhütung
BI: behavioral intention
EE: effort expectancy
FC: facilitating condition
HLC: health locus of control
HM: hedonic motivation
HT: habit
LOC: locus of control
MCI: Management Center Innsbruck
PE: performance expectancy
PS: price sensitivity
SI: social influence
UTAUT2: Unified Theory of Acceptance and Use of Technology-2
VRodel: virtual reality sledding game for safety education
