# Supplementary material for: Parents’, Teachers’, and Sledders’ Acceptability of a Virtual Reality Game for Sledding Safety Education: Cross-Sectional Study
Source: JMIR Form Res. 2025 May 21;9:e63813. doi: 10.2196/63813 (PMC12118773; doi:10.2196/63813)
Supplement: Multimedia Appendix 1 [file formative-v9-e63813-s001.docx]

**Supplemental online material 1**

**Original German version description of the virtual reality sledding game for safety education**

Das Spiel „VRodel“ wird zukünftig einen Rodel Simulator darstellen, welcher auf der Nutzung der virtuellen Realität (VR) basiert und beliebte Rodelbahnen in Tirol realitätsgetreu nachbildet. In diesem Spiel sollen Kinder die Möglichkeit besitzen, spielerisch Sicherheitskompetenzen für das Rodeln zu entwickeln. Das Spiel läuft auf einem Computer während dem/r Anwender/in die Spielumgebung auf einer VR Brille dargestellt wird. Gleichzeitig erfolgt die Steuerung im Spiel mittels je eines Controllers pro Hand. Die Kinder können dabei innerhalb des Spiels den Spaß am Rodeln sicher und realistisch in der virtuellen Welt erleben, ohne den Gefahren von Verletzungen und Wettereinflüssen ausgesetzt zu sein. In der Anwendung sollen die Kinder Fähigkeiten wie die Steuerung des Rodels, die Kontrolle über ihre Geschwindigkeit sowie das richtige Befahren von Kurven in möglichst realistischen Szenarien kennenlernen. Um dieses Spiel möglichst bedarfsgerecht zu entwickeln und eine zukünftige Nutzung durch Kinder (unter Anleitung von Eltern oder Lehkräften) zu gewährleisten, würden wir gerne Ihre Meinung zu dieser neuen Anwendung namens „VRodel“ erfahren.


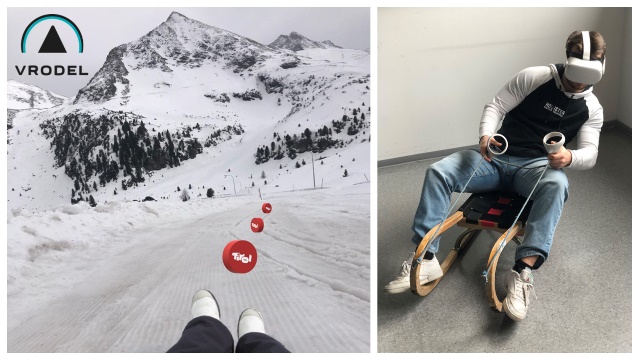


*Schematische Darstellung eines "VRodel" Spielers sowie eines möglichen Spielausschnittes*

**Translated English version** **description of the virtual reality sledding game for safety education**

The game “VRodel” will be a sledding simulator based on virtual reality (VR) and will realistically replicate popular sledding runs in Tyrol. In this game, children will have the opportunity to playfully develop safety skills for sledding. The game runs on a computer while the user is shown the game environment on VR glasses. At the same time, the game is controlled by one controller per hand. Within the game, children can experience the fun of sledding realistically and safely in the virtual world, without being exposed to the dangers of injuries and weather conditions. In the application, the children should learn skills such as how to control the toboggan, control their speed and negotiate bends correctly in scenarios that are as realistic as possible. In order to develop this game to meet the needs as much as possible and to ensure future use by children (under the guidance of parents or teachers), we would like to know your opinion about this new application called "VRodel".


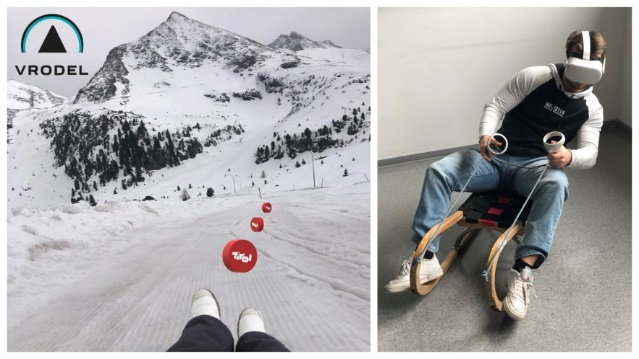


*Schematic representation of a "VRodel" player and a possible example of the game.*
