# Supplementary material for: Parents’, Teachers’, and Sledders’ Acceptability of a Virtual Reality Game for Sledding Safety Education: Cross-Sectional Study
Source: JMIR Form Res. 2025 May 21;9:e63813. doi: 10.2196/63813 (PMC12118773; doi:10.2196/63813)
Supplement: Checklist 1 [file formative-v9-e63813-s002.pdf]

RATE-XR checklist (Vlake et al., 2024)
JFR ms#63813

Theme	Recommendation	Lines in the manuscript
Title	*Identify the study as an early clinical evaluation, or a similar term, of an application using XR[b], or a more specific term, in the title, including its intended aim.*	2-3
**Abstract**	Provide a (structured) summary of the study.<br>Consider including the following:<br>• A concise description of the clinical problem or knowledge gap and the rationale for using an application using XR<br>• A concise description of the study methods, including a short description of the application including its name, study population, study setting, main outcomes, and assessment methods.<br>• A concise description of the results, including safety and harm outcomes<br>• A short conclusion<br>• If applicable, details about the registration of the study in a publicly available database.	5-39
**Introduction**		
Clinical problem and existing	*Introduce the clinical problem for which the application using XR was used, including its relevance and a description of (the efficacy of) evidence-based or commonly used interventions or the treatment as usual, which is intended to be replaced by the application using XR.*	69-73
Introduction of the application	*Introduce the application using XR, including the following:*<br>• *Hypotheses for the potential effect; how the application is expected to contribute to the clinical problem.*<br>• *If available, a concise description of, or a reference to, previous research on the same (or a similar) application.*	N.A.
Objectives	Specify the study objectives or hypotheses.	184-206
**Methods and analysis**		
Trial design and reporting	Provide a reference to ethical approval and, if available, to any (published) study protocol and registration of the study in a publicly available repository.	209-213
Trial design and reporting	Describe, and mention the rationale for, the study design. For clarification, it is recommended to use a flow diagram.	215-219
Participants and setting	*Describe the setting and locations, including country, where data were collected and processed and where the application using XR was applied and evaluated.*	209-232; supplementary 1
Participants and setting	*Describe how participants were selected and recruited and provide eligibility criteria.*	22-232
Participants and setting	*Describe who will be applying the application and whether they were trained.*	N.A.
Intervention and procedures	*Provide a description of the application, including its content, hardware, protocol, and set-up, or provide a reference to previous publications where this information is described. Consider supplementing the description with an image, figure, or film.*	supplementary 1
Intervention and procedures	*Describe, or provide a reference to, the development process of the application.*	N.A.
Intervention and procedures	*Describe the participant timeline in sufficient detail to allow replication, including all procedures, co-interventions (if applicable), and (follow-up) assessments.*	208-342
Intervention and procedures	Describe and give a rationale for the control conditions or provide a rationale for not using one.	N.A.
Outcomes	Describe all prespecified primary and secondary outcomes, including how and when assessed.	208-342
Outcomes	*Describe how safety and harm outcomes were assessed. Describe which, and how, other XR specific outcomes were assessed, such as performance, usability, presence, perspectives, and acceptability.*	208-342
Sample size	Provide a justification for the sample size.	339-342
Analysis	Provide a detailed description of how primary and secondary outcomes were analyzed, including any prespecified comparisons or stratifications.	312-338
Protocol alterations	Describe changes to the methods or protocol, including procedures, study outcomes, eligibility criteria, and analysis plan, after study commencement, with reasons, and, if applicable, report whether the study registration was updated.	N.A.
Results		
Participant flow and recruitment	Describe the time frame of recruitment and follow-up and the participant flow, including the number of patients screened and included, receiving the intervention, and being included in each analysis. Report if, and why, the study was prematurely terminated. The use of a flow diagram is highly recommended.	344-354
Baseline data	Describe, or add a table depicting, baseline and treatment-related characteristics. If applicable, describe and specify any concurrent measures.	Table 1

Main results	Report on all prespecified outcomes that are available. Consider using tables, figures, or graphs to illustrate results.	357-426
XR and human factors	*Include information about the usage of the application, such as duration, frequency, number of sessions, error rates, and number of sessions requiring interruption or discontinuation, including reasons.*	N.A.
XR and human factors	*If assessed, report on XR-specific outcomes, such as performance, usability, presence, perspectives, and acceptability.*	357-426
Safety and harms	*Report on safety and harms, including unintended effects, both during and after using the application.*	N.A.
Discussion and conclusion		
Generalizability and impact	Discuss (potential) impact of study findings and generalizability, including barriers for the use and implementation of the application.	466-473; 491-499
Safety and harms	Discuss safety and instances of harm, including their possible effects on study findings, implications for future use of the applications, and whether they can be prevented or mitigated.	N.A.
Ethics	Describe ethical considerations, including benefits and risks, for the current and future use of the application.	N.A.
Strengths and limitations	Discuss study strengths and limitations, including sources of potential bias.	475-499
Conclusion	*Provide a conclusion that accurately interprets study findings, including future perspectives.*	533-542
Statements		
Funding and conflicts of interest	Disclose any potential conflict of interest, real or apparent, including the funding sources and their roles in the design, conduct, analysis, and report of the study, potential roles of commercial companies, and personal conflicts of interest for each author.	544-563
Application	*Indicate whether the application is a commercial product, it is publicly available, it can be accessed, it complies with the medical device regulations, and whether the application was approved for its intended use by a formal regulatory body or if the study is part of the clinical evaluation for future certification.*	N.A.

NA. Not applicable as there is no actual use of the application, since this is an acceptability study based on a hypothetical description of the virtual reality game.
